# Comparison of long-term outcomes of young patients after a coronary event associated with familial hypercholesterolemia

**DOI:** 10.1186/s12944-019-1074-8

**Published:** 2019-06-01

**Authors:** Xu Wang, Gaojun Cai, Yingying Wang, Ran Liu, Ziwei Xi, Gexuan Li, Wenhui Wen, Yue Wu, Chenggang Wang, Qingwei Ji, Xinguo Wang, Qian Zhang, Yujie Zeng, Luya Wang, Wei Liu, Yujie Zhou

**Affiliations:** 1 Emergency & Critical Care Center, Beijing Anzhen Hospital, Capital Medical University, Beijing, 100029 China; 2 Department of Cardiology, 12th ward, Beijing Anzhen Hospital, Capital Medical University, Beijing Institute of Heart Lung and Blood Vessel Disease, Beijing Key Laboratory of Precision Medicine of Coronary Atherosclerotic Disease, Clinical center for coronary heart disease, Capital Medical University, Beijing, 100029 China; 3 Beijing Institute of Heart Lung and Blood Vessel Disease, the Key Laboratory of Remodeling-related Cardiovascular Disease, Ministry of Education, Beijing Anzhen Hospital, Capital Medical University, Beijing, 100029 China; 4 Department of Cardiology, Wujin Hospital affiliated with Jiangsu University, Changzhou, 213017 Jiangsu China; 5 Department of Emergency, Tongliao Hospital, Tongliao, 028000 Inner Mongolia China

**Keywords:** Familial hypercholesterolemia, Coronary event, Outcomes

## Abstract

**Objective:**

Familial hypercholesterolemia (FH) is an important cause of premature coronary artery disease (CAD). Prognosis data are lacking in patients with FH and coronary artery disease particularly in the era of widespread statin use. We compared long-term prognosis between patients with and without FH after a coronary event.

**Methods:**

In this retrospective study, 865 patients younger than 40 years of age with CAD were enrolled. FH was diagnosed based on the Dutch Lipid Clinic Network algorithm. Baseline characteristics, coronary angiographic findings and prognosis during median follow-up of 5 (3–8) years were compared between patients with or without FH.

**Results:**

Definite or probable FH was detected in 37 patients (4.3%) and possible FH in 259 patients (29.9%). FH was associated with significantly higher prevalence of multi-vessel lesions (*p* < 0.001) and higher Gensini score (*p* = 0.008). In the subset of 706 patients for whom follow-up data were available, 127 (18.0%) suffered major adverse cardiovascular and cerebrovascular events (MACCE). FH was associated with increased risk of MACCE, independently of age, sex, smoking, body mass index, hypertension or diabetes mellitus (HR = 2.30, 95%CI = 1.09 to 4.84, *p* = 0.028).

**Conclusions:**

FH is an independent risk factor for MACCE in young patients after a coronary event during long-term follow-up. It is necessary to optimize lipid treatment of patients with FH after a coronary event.

## Introduction

Familial hypercholesterolemia (FH; MIM 143890) is an autosomal dominant genetic disease characterized by high levels of low-density lipoprotein cholesterol (LDL-C), cutaneous xanthomas and premature coronary artery disease (pCAD), which has recently become a major health concern around the world [1]. Data suggest that 1 in 200 of Caucasians are heterozygous for FH and that 1 in 160,000–300,000 are homozygous [2], which are much higher prevalences than those estimated a decade earlier [3]. A study in China has suggested a prevalence of probable or definite FH of 0.28% (1.4/500) based on the modified Dutch Lipid Clinic Network (DLCN) definition [4]. These studies suggest that nearly 36 million people around the world have potential FH, including 3.8 million in China. FH remains largely underdiagnosed and undertreated. Fewer than 1% of FH cases are diagnosed in most countries, except in some countries of western Europe [2].

Timely detection of FH is important because the presence of definite or probable FH increases 13-fold (95%CI = 10–17) risk of CAD compared to the general population [5]. In Europe, FH occurs in up to 8% of patients admitted for acute coronary syndromes, a frequency that is 10 times higher than in the general population [6]. For patients with acute coronary syndromes, the diagnosis of FH during hospitalization helps to ensure the use of high-dose statins and the management of blood lipids after discharge [7].

Whether FH in CAD alters the clinical manifestations of the disease and the prognosis of patients is unclear. We compared the long-term prognosis of young patients with and without FH after a coronary event.

## Methods

### Study population

Patients with both gender (≥18 years and < 40 years of age) with the following first or recurrent clinical diagnoses or procedures at Beijing Anzhen Hospital from January 2004 to January 2015 were consecutively enrolled in this retrospective study, including those with elective or primary PCI, elective or emergency CABG, acute myocardial infarction (AMI: ICD-10 I21), and acute myocardial ischemia (ICD-10 I20) [8]. The diagnosis of every participate enrolled in this study was confirmed by two experienced cardiologists.

Patients were excluded if blood lipid data were missing or if the patients had infectious or systematic inflammatory disease, significant hematologic disorders, thyroid dysfunction, severe liver and/or renal dysfunction, or malignant tumors. The study was approved by the Institutional Ethics Committee of Beijing Anzhen Hospital. The Ethics Committee waived the requirement for informed consent because of the retrospective nature of the study.

Patients enrolled in the study were diagnosed with FH based on the DLCN criteria (Table 1), which are endorsed by ESC/EAS guidelines [9]. Patients were classified as having possible FH if their DLCN score was 3–5, “definite” FH was > 8, “probable” FH was 6~8 [10]. In our study, we combined the categories “definite” FH and “probable” FH into one category (definite/probable FH). Family history of elevated LDL-C was not available for our study sample, so this information was counted as ‘0’ in the DLCN algorithm. Similarly, genetic tests of FH were not conducted and were counted as ‘0’.

**Table 1:** Dutch Lipid Clinic Network criteria for diagnosis of heterozygous familial hypercholesterolaemia in adults

Criteria	Points
Family History	
A First degree relative with known premature (< 55 years men; < 60 years women) coronary disease and vascular disease OR LDL-c > 95th percentile	1
B First degree relative with tendon xanthomata and/or arcuscornealis OR childhood (< 18 years) with LDL-c > 95th percentile	2
Clinical history	
A Patient with premature CAD (men <55, women <60 years)	2
B Patient with premature cerebral or PVD (men <55, women <60 years)	1
Physical Examination	
A Tendon xanthomas	6
B Premature arcus	4
biochemical results (LDL cholesterol)	
A LDL – c > 8.5 mmol/L	8
B LDL-c 6.5–8.4 mmol/L	5
C LDL- c 5.0–6.4 mmol/L	3
D LDL – c 4.0–4.9 mmol/L	1
molecular genetic testing (DNA analysis)	
DNA mutations	8
Definite FH: > 8 points, Probable FH: 6–8 points, Possible FH: 3–5	

### Patient assessment

Data on clinical characteristics were collected based on review of medical records or patient interviews. All patients underwent clinical examination, blood testing and angiography. Peripheral blood samples were drawn from patients within 24 h after admission, and lipid profiles and other assays were performed using an automated biochemical analyzer. LDL-cholesterol concentrations were multiplied by 1.43 in individuals using cholesterol lowering medication before admission [11]. Hypertension was defined according to the 2010 Hypertension Prevention and Treatment Guidelines [12] as systolic blood pressure ≥ 140 mmHg and/or diastolic blood pressure ≥ 90 mmHg or the taking of antihypertensive medication. Diabetes mellitus was defined according to the American Diabetes Association (ADA) Diabetes Diagnostic Criteria [13]. Data were collected on smoking status and body mass index (BMI). After release from hospital, patients were followed up through phone interviews, review of hospital records or outpatient visits.

The primary outcome was main adverse cardiovascular and cerebrovascular events (MACCE), which were defined as cardiac death, acute myocardial infarction, acute decompensated heart failure requiring hospitalization, cerebrovascular events or ischemia-driven revascularization. Ischemia-driven revascularization was defined as repeated PCI or CABG of lesions in the presence of acute myocardial infarction, unstable or stable angina, or documented silent ischemia.

### Statistical analysis

Continuous variables were presented as the mean ± standard deviation (SD) if the data were normally distributed. Otherwise, the data were presented as median and interquartile range (IQR). Categorical variables were expressed as frequencies and percentages. Differences in clinical characteristics between patients with or without FH were assessed for significance using one-way ANOVA, χ^2^ tests or the Kruskal-Wallis rank test. Hazard ratios (HRs) and corresponding 95% confidence intervals (CIs) were calculated using Cox proportional hazard models. Event-free survival was analyzed using the Kaplan-Meier method, and inter-group differences in survival were assessed for significance using the log-rank test. All statistical calculations were performed in SPSS 22 (IBM, Chicago, IL, USA), with *p* < 0.05 defined as indicating significance.

## Results

### Baseline characteristics

Among 865 participants, 37 (4.3%) had definite/probable FH and 259 (29.9%) had possible FH, respectively. The remaining 569 (65.8%) received DLCN scores of “unlikely FH” and so were classified as not having FH (Table 2). Unstable angina pectoris is the main cause of hospitalization in FH patients (56.8%), while ST-segment elevation myocardial infarction (STEMI) is a common cause of hospitalization in non-FH patients (57.3%). Compared with patients without FH, those with definite or probable FH were younger but showed higher prevalence of multi-vessel disease (75.7% vs 34.1%), chronic total occlusion (CTO) (45.9% vs 29.9%)and greater coronary severity based on Gensini score (50 vs 32). More patients with definite or probable FH received bypass grafting than those without FH (32.4% vs 10.7%) (Table 3). When discharge, 75.7% of patients with definite or probable FH were on statin therapy, 24.3% on a combination of statin and ezetimibe, and 5.4% on high-dose statin.

**Table 2:** Baseline characteristics of young patients after a coronary event with or without FH

Variable	Total	FH status^a^			
		Probable/Definite (> 5 points)	Possible (3–5 points)	None (< 3 points)	*P*
Number, n (%)	865 (100)	37 (4.3)	259 (29.9)	569 (65.8)	
Demographics					
Age, yr	33 (30–34)	31 (28–31)	33 (31–35)	33 (30–34)	0.005
Female, n(%)	49 (5.7)	2 (5.4)	15 (5.8)	32 (5.6)	0.993
BMI, kg/m^2^	27.8 (4.09)	26.5 (4.58)	28.3 (3.82)	27.7 (4.15)	0.057
Family history of pCAD, n(%)	199 (23.0)	21 (56.8)	178 (68.7)	0 (0.0)	0.000
Smoking status, n(%)	586 (67.7)	28 (75.7)	177 (68.3)	381 (67.0)	0.531
Elevated alcohol consumption, n(%)	208 (24.0)	9 (24.3)	55 (21.1)	144 (25.3)	0.445
Past history					
Hypertension, n(%)	318 (36.8)	7 (18.9)	110 (42.5)	201 (35.3)	0.013
Diabetes mellitus, n(%)	138 (16.0)	2 (5.4)	46 (18.0)	90 (15.7)	0.145
Pre-existing CAD, n(%)	162 (18.7)	8 (21.6)	45 (17.6)	109 (19.1)	0.792
Lipid profiles					
TC, mmol/L	4.58 (1.46)	8.10 (2.35)	5.02 (1.33)	4.15 (1.00)	0.000
LDL-C, mmol/L	2.92 (1.24)	6.37 (2.13)	3.30 (1.12)	2.53 (0.71)	0.000
HDL-C, mmol/L	0.90 (0.20)	0.91 (0.19)	0.91 (0.21)	0.89 (0.20)	0.472
TG, mmol/L	1.90 (1.29–2.78)	1.56 (1.11–2.55)	2.01 (1.50–2.98)	1.83 (1.27–1.74)	0.017

*FH* familial hypercholesterolemia, *pCAD* premature coronary artery disease, *BMI* body mass index, *TC* total cholesterol, *LDL-C* low-density lipoprotein cholesterol, *HDL-C* low-density lipoprotein cholesterol, *TG* triglyceride

^a^Based on Dutch Lipid Clinic Network criteria

**Table 3:** Clinical features, angiographic characteristics, and treatment of young patients after a coronary event with or without FH

Variable	Total	FH status^a^			
		Probable/Definite (> 5 points)	Possible (3–5 points)	None (< 3 points)	*P*
Diagnosis					
STEMI, n(%)	468 (54.1)	16 (43.2)	126 (48.6)	326 (57.3)	0.027
NONSTEMI, n(%)	75 (8.7)	0 (0.0)	29 (11.2)	46 (8.1)	0.054
UAP, n(%)	314 (36.3)	21 (56.8)	101 (39.0)	192 (33.7)	0.010
SAP, n(%)	8 (0.9)	0 (0.0)	3 (1.2)	5 (0.9)	0.774
Coronary angiography					
None, n(%)	100 (11.6)	1 (2.7)	33 (12.7)	66 (11.6)	0.216
Single vessel, n(%)	448 (51.8)	8 (21.6)	131 (50.6)	309 (54.3)	0.001
Double vessel, n(%)	195 (22.5)	13 (35.1)	57 (22.3)	125 (21.9)	0.173
Triple vessel, n(%)	110 (12.7)	13 (35.1)	34 (13.1)	63 (11.1)	0.000
Left main, n(%)	61 (7.1)	3 (8.1)	13 (5.0)	45 (7.9)	0.312
CTO, n(%)	270 (31.2)	17 (45.9)	83 (32.2)	170 (29.9)	0.115
Multivessel lesion, n (%)	317 (36.6)	28 (75.7)	95 (36.7)	194 (34.1)	0.000
Gensini	32 (10–64)	50 (14–82)	32 (10–64)	32 (9–56)	0.008
Operation					
PCI, n(%)	539 (62.3)	20 (54.1)	176 (68.0)	343 (60.3)	0.061
CABG, n(%)	90 (10.4)	12 (32.4)	17 (6.6)	61 (10.7)	0.000
PTCA, n(%)	20 (2.3)	0 (0.0)	6 (2.3)	14 (2.5)	0.628
Medication at discharge					
Aspirin, n(%)	819 (94.7)	36 (97.3)	251 (96.9)	532 (93.5)	0.098
β-blocker, n(%)	701 (81.0)	26 (70.3)	216 (83.4)	459 (80.7)	0.151
ACEI/ARB, n(%)	472 (54.6)	13 (35.1)	146 (56.4)	313 (55.0)	0.049
Statins, n(%)	719 (83.1)	28 (75.7)	226 (87.3)	465 (81.7)	0.067
High-dose statins, n(%)	67 (7.7)	2 (5.4)	21 (8.1)	44 (7.7)	0.847
Ezetimibe, n(%)	40 (4.6)	9 (24.3)	15 (5.8)	16 (2.8)	0.000

*FH* familial hypercholesterolemia, *pCAD* premature coronary artery disease, *STEMI* ST-segment elevation myocardial infarction, *NONSTEMI* Non-ST-segment elevation myocardial infarction, *UAP* Unstable angina pectoris, *SAP* Stable angina pectoris, *CTO* chronic total occlusion, *CABG* coronary artery bypass grafting, *PCI* percutaneous coronary intervention, *PTCA* percutaneous transluminal coronary angioplast, *ACEI/ARB* angiotensin converting enzyme inhibitors/angiotensin receptor blocker

^a^Based on Dutch Lipid Clinic Network criteria

### The association between FH and risk of MACCE

Follow-up data were available for 708 (81.8%) patients, while the remaining patients were lost to follow-up, such as because they moved or changed their telephone number. During follow-up, which lasted for a median of 5 (3–8) years, one patient without FH died of leukemia and one without FH died of a brain tumor. These two patients were excluded from the follow-up analysis. Among the remaining 706 follow-up patients, 127 (18.0% of all patients followed-up) experienced a cardiovascular event, which was myocardial infarction in 36 cases (5.1% of all patients followed-up) and cardiac deaths in 15 cases (2.1% of all patients followed-up), respectively. In an unadjusted model, patients with definite or probable FH were at 2.34-fold higher risk of MACCE than those without FH (HR = 2.34, 95%CI = 1.17–4.68, *p* = 0.016). In a multivariable model adjusted for age and sex, patients with definite or probable FH were at 2.39-fold higher risk of MACCE than patients without FH (HR = 2.39, 95%CI = 1.19–4.78, *p* = 0.014). These HR estimates did not decrease when the multivariable model also adjusted for BMI, smoking, hypertension, and diabetes mellitus (HR = 2.30, 95%CI = 1.09–4.84, *p* = 0.028) (Table 4).

**Table 4:** Multivariable analysis of the association between FH and risk of MACCE

FH status^a^	Participants (n)	MACCE (n)	Incidence rate (per 100 person-y)	Unadjusted HR (95% CI)	Model 1 HR (95% CI)	Model 2 HR (95% CI)
No FH (Score < 3)	453	73	2.5	1.00 (Referent)	1.00 (Referent)	1.00 (Referent)
Possible FH (Score 3–5)	223	45	3.3	1.30 (0.89–1.88)	1.30 (0.90–1.89)	1.16 (0.77–1.75)
Probable/Definite FH (Score > 5)	30	9	5.8	2.34 (1.17–4.68)	2.39 (1.19–4.78)	2.30 (1.09–4.84)

*FH* familial hypercholesterolemia, *MACCE* major adverse cardiovascular and cerebrovascular events, *HR* hazard ratio, *CI* confidence interval. Model 1, adjusted for age and sex. Model 2, adjusted for age, sex and cardiovascular risk factors, including current smoking, hypertension, diabetes mellitus, and BMI

^a^Based on Dutch Lipid Clinic Network criteria

## Discussion

This study suggests that the prevalence of definite or probable FH among young patients after a coronary event was 4.3%, while that of possible FH was 29.9%, based on the DLCN algorithm. In our cohort, FH was associated with more serious coronary lesions and greater prevalence of multi-vessel disease, despite being associated with younger age. FH was independently associated with increased risk of MACCE during long-term follow-up. Our study suggests that timely diagnosis of FH may help stratify the risk of future MACCE in young patients after a coronary event, as well as identify patients whose close relatives should be screened.

Due to lack of national data, we cannot compare our measured prevalence of 4.3% for definite or probable FH in our cohort with statistics for the population of CAD patients or the general population in China. A single-center study in China reported a prevalence of 3.9% for definite or probable FH in patients with acute myocardial infarction and 7.1% in patients with premature myocardial infarction [14]. A European multi-country study involving 1451 patients with premature acute coronary syndrome and younger than 55 years (men) or 60 years (women) reported prevalence of 4.8% for definite or probable FH and 47.1% for possible FH [6].

As a hereditary disease, the clinical diagnostic criteria of FH can identify FH rapidly and costly. We found that patients with definite or probable FH, despite their younger age, exhibited more severe coronary atherosclerosis and higher prevalence (75.7%) of multi-vessel lesions than patients without FH (34.1%). Patients with FH tend to receive lipid-lowering treatment relatively late, when severe atherosclerosis has developed, and the efficacy of lipid-lowering therapy may be reduced. In addition, we found that few of patients with FH in this study were discharged on high-dose statin therapy. Instead, our clinicians preferred to use moderate doses of statins on their own or combined with ezetimibe or bile acid sequestrants in order to enhance lipid-lowering therapy while minimizing the statin-associated risk of elevated liver enzymes and creatine kinase. Statin monotherapy fails to achieve optimal LDL-C levels in most patients with FH [15]. More effective lipid-lowering strategies include high-dose statin in combination with evolocumab [16, 17] or ezetimibe. Bile acid sequestrants are rarely used because of their adverse gastrointestinal side effects and poor patient compliance. Colesevelam has stronger ability to bind bile acid. Monotherapy with colesevelam reduced LDL cholesterol by 13–19% and additional 18% in combination with statins [18]. While treatment for CAD patients with FH remains to be optimized, statins can substantially improve prognosis: modest doses of statins can reduce risk of CAD by about 80% in patients with FH [19], and a prospective registry study involving 3382 patients with FH showed that statins can reduce coronary mortality by 30% [20].

With regard to clinical outcomes, we found that definite or probable FH had a > 2-fold increase MACCE during a long-term follow-up comparing with the patients without FH. This association between FH clinical diagnosis and MACCE was independent of conventional risk factors. Since the lipid-lowering therapy in patients with FH is generally quite late, the coronary artery lesions in patients with FH are more serious. This may be one of the reasons for the adverse outcomes in these patients. In addition, even after a coronary event, the rate of high-dose statin therapy in FH patients was fairly inadequate in this cohort. These patients were still exposed to high levels of LDL-C. Furthermore, though some patients with FH were treated with high-dose statin combined with ezetimibe, part of them still could not attain the desirable targets of LDL-C levels. For these patients, novel lipid-lowering drugs such as proprotein convertase subtilisin kexin 9 inhibitors are expected to further decrease the LDL-C levels and improve cardiovascular outcomes [21, 22].

In conclusion, early diagnosis and treatment are crucial for improving the outcomes of FH patients. Screening for FH may allow timelier and therefore more effective lipid management in CAD patients with FH. It may also identify patients whose close family members should be screened for FH in order to ensure timely intervention to prevent cardiovascular events.

### Limitations

The results of this study should be interpreted with caution in light of several limitations. One is the retrospective design, although all patients were followed up prospectively. Secondly, we did not perform genetic molecular analysis to identify heterozygous FH patients. In addition, not all patients received similar treatment. Finally, family history of elevated LDL-C was not available for the presents study, which might underestimate the prevalence of FH in this population.

## Conclusions

It is necessary to optimize lipid treatment of patients with FH after a coronary event. This study provides evidence that FH is an independent risk factor for long-term MACCE in young patients after a coronary event.

## Data Availability

The datasets used and/or analyzed during the current study will be available from the corresponding author on reasonable requests after study completion.

## Abbreviations

ACEI/ARB: angiotensin converting enzyme inhibitors/angiotensin receptor blocker
BMI: body mass index
CABG: coronary artery bypass grafting
CAD: Coronary artery disease
CTO: chronic total occlusion
FH: Familial hypercholesterolemia
HDL-C: High density lipoprotein cholesterol
LDL-C: low-density lipoprotein cholesterol
MACCE: major adverse cardiovascular and cerebrovascular events
NONSTEMI: Non-ST-segment elevation myocardial infarction
pCAD: Premature coronary artery disease
PCI: percutaneous coronary intervention
PTCA: percutaneous transluminal coronary angioplast
SAP: Stable angina pectoris
STEMI: ST-segment elevation myocardial infarction
TC: Total cholesterol
TG: Triglyceride
UAP: Unstable angina pectoris
